# The Effect of Potential on Surface Characteristic and Corrosion Resistance of Anodic Oxide Film Formed on Commercial Pure Titanium at the Potentiodynamic-Aging Mode

**DOI:** 10.3390/ma12030370

**Published:** 2019-01-24

**Authors:** Ling Zhang, Yanqing Duan, Rui Gao, Jianyun Yang, Keyi Wei, Danyu Tang, Tianlin Fu

**Affiliations:** 1 R&D Center of China Tabacco Yunnan Industrial Co., Ltd., Yunnan 650000, China; zhangling@ynzy-tobacco.com (L.Z.); duanyq@ynzy-tobacco.com (Y.D.); zhang-874005@163.com (R.G.); zzy9939@163.com (J.Y.); keyiwei@126.com (K.W.); tangdgirl@126.com (D.T.);; 2 School of Materials Science and Engineering, South China University of Technology, Guangzhou 510641, China

**Keywords:** commercially pure titanium, passive film, surface characteristic, potentiodynamic-aging mode, corrosion resistance

## Abstract

Anodic oxidation treatment of commercially pure titanium was carried out at the voltages of 10, 30, 50 V in 0.5 M H_2_SO_4_ solution at the potentiodynamic-aging mode so as to obtain the effects of the anodic potential on the surface characteristic and corrosion resistance of the anodic oxide film. The influences of potential on the surface morphology, the roughness, the crystalline behavior, the chemical composition and the corrosion resistance of the anodic oxide films were investigated by using scanning electron microscopy (SEM), atomic force microscope (AFM), Raman spectrum, X-ray diffractometry (XRD), X-ray photoelectron spectroscopy (XPS), potentiodynamic polarization curves and electrode impedance spectroscopy (EIS). The results show that increasing anodic potential at the potentiodynamic-aging mode can significantly enhance thickness, flatness, crystallization, chemical stability, and corrosion resistance of anodic oxide film.

## 1. Introduction

Titanium and its alloys have been extensively used in aerospace, marine engineering, biomedical applications due to their excellent properties [1]. The excellent corrosion resistance of titanium is due to an ultra-thin amorphous film, which grows spontaneously on its surface in air and aqueous environments (Ti + O_2_ = TiO_2_, ΔG_0_ = −888.8 kJ·mol^−1^) [2]. This native oxide film can prevent the metal from further reacting with corrosive environments [3]. Unfortunately, these films have many defects, only a few nm in thickness [4]. What is more, according to Hanawa et al. [5], the oxide film formed in air is composed of Ti^4+^, Ti^3+^, Ti^2+^ and metallic Ti^0^. Multivalent Ti is thermodynamically less favorable than Ti^4+^. As a result, it can be destroyed easily owing to many reasons, leading to crevice and galvanic corrosion [4,6].

Surface modification methods are extensively applied in improving corrosion resistance of the titanium and its alloys, such as plasma, ion implantation, anodic oxidation treatment, spraying, etc. [7,8]. Among them, anodic oxidation treatment is one of the most important surface modification techniques on account of its lower cost and operability [9]. The corrosion resistance of the anodic oxide film depends on its morphology, thickness, chemical composition, and crystallization. In addition, these properties can be programmed by using a suitable electrolyte, anodic density, anodic potential, treated time and anodic modes. 

Anodic potential may be the most important parameter for the anodic oxide film. Diamanti et al. [1] revealed that high potentials bring about a crystalline structure of the anodic films on titanium, on the contrary, low potentials lead to an amorphous structure. Karambakhsh et al. [10] reported that the thickness was increased by the ascending anodic potential. The crystalline titanium oxide film is more stable than the amorphous one, and the corrosion resistance increases with thickness of the oxidic film [11]. Therefore, increasing the anodic potential is beneficial to enhance the corrosion resistance of titanium and its alloy. However, several studies have also shown that the higher anodic potential can result in the increase of the roughness and the formation of micropores on the surface of Ti and its alloy [12,13]. As is known to all, a material with a flat and dense surface may have a better corrosion resistance [14]. Sivaet al. [15] studied the effect of surface roughness of industrial pure titanium on corrosion resistance, and electrochemical tests revealed the lower the surface roughness of industrial pure titanium, the better its corrosion resistance. That is because the aggressive ions can be preferentially adsorbed into the pores and cracks of the anodic oxide films, which may cause localized corrosion.

In order to further enhance the corrosion resistance of the anodic oxide film, it is necessary to reduce its surface roughness and porosity while improving its thickness and crystalline. According to literature [16,17], the properties of the anodic oxide films are considerably influenced by the anodization mode, even if the final anodic potential has a constant value. There are mainly three anodization modes, including potentiostatic mode, galvanostatic mode, and potentiodynamic mode. The anodic oxide film formed on potentiostatic mode is thicker and more crystalline than the potentiodynamic mode and galvanostatic mode films [18,19]. However, Xing et al. [17] also reported that the potentiodynamic grown film is smoother than the film formed at the potentiostatically mode.

From these previous results, it can be concluded that potentiostatically mode or potentiodynamic mode has its own advantage. Therefore, the purpose of this study to improve the thickness and the crystallinity, while reducing the roughness and improving the surface compactness of the oxide film by an anodization mode named potentiodynamic-aging mode. To our best knowledge, no systematic study has been done to evaluate potentiodynamic-aging mode on the properties of commercial pure titanium (CP-Ti). The goal is to understand the effect of potential on surface characteristic and corrosion resistance of anodic oxide film formed on CP-Ti at the potentiodynamic-aging mode. The surface topography, crystalline, thickness, and composition of CP-Ti oxide films at the potentiodynamic-aging mode as a function of anodic potential in 0.5 M H_2_SO_4_ solution are studied. The electrochemical tests were conducted in 3.5 wt.% NaCl solution by means of electrochemical impedance spectroscopy (EIS) and potentiodynamic polarization curves. Electrochemical data were correlated to anodic oxide film composition. In addition, the film growing and crystallization process of anodic oxide films on CP-Ti under potentiodynamic-aging mode are discussed.

## 2. Experimental

### 2.1. Specimen Preparation

The nominal composition of industrial pure Ti used in this research was (wt.%) C 0.02, N 0.02, Fe 0.10, O 0.15, H 0.0011, and the rest is Ti. The test-pieces were cut into sizes of 50 × 10 × 1 mm^3^, cleaned with acetone, alcohol and deionized water under the ultrasonic condition. Subsequently, the titanium sheets were etched using Kroll’s reagent (1 mL HF and 5 mL HNO_3_ in 44 mL H_2_O) for 10 min [20], and then rinsed with deionized water for 20 min under the ultrasonic condition again. Anodic oxide films were formed in a two-electrode electrochemical cell. Using the test-piece as the anode, a graphite plate as the cathode. The anode and cathode were positioned face-to-face with a 10 mm distance in 0.5 M H_2_SO_4_. During anodic treatment, the potential was firstly swept from 0 to 10, 30 or 50 V at a constant sweep rate of 1 V/s, and then was kept at the final potential for 3600 s. All experiments were carried out at room temperature. The final samples were termed Ti_x_, where x = 10, 30 or 50.

### 2.2. Surface Characterization

The micromorphology of the sample was observed using scanning electron microscopy (SEM, LEO 1530Vp, Carl Zeiss AG, Heidenheim, Germany) and the acceleration voltage was 15 kV. The 3D morphology of passive film was characterized by atomic force microscope (AFM, CSPM-400, China Benyuan nano co., Ltd., Beijing, China). The Si probe tips (force constant: 3.0 N/m) used for the AFM measurements were integral with the V-shaped cantilevers. A Raman spectrometer equipped with an optical microscopy (LabRAM HR Evolution, Horiba Group, Kyoto, Japan) was used to detect the crystallization of anodic oxide films on titanium. The excitation wavelength was 532 nm, and the incident power was 10.4 mW. To eliminate influence on the values of binding energies as a result of the X-ray photoelectron spectroscopy (XPS, PHI5000 Versaprobe-II, Shimadzu co., Ltd., Kyoto, Japan) analysis may charge the sample, all data were corrected by a linear shift so as to the peak maximum of the C 1s originated from adventitious carbon corresponded to 284.8 eV [21]. An XPS analysis software named Multipak (Shimadzu co., Ltd., Kyoto, Japan) was used to fit the existing forms of Ti and O in passive films. The quantitative elemental analysis was performed determining peak areas and taking into account empirical sensitivity factors for each element. To analyze the thickness and the elemental contents longitudinal changes of the passive film, a 3000 eV Ar^+^ ion beam sputtered on the surface over an area of 4 × 4 mm^2^.

### 2.3. Corrosion Behavior

The corrosion resistance of samples was assessed through electrochemical tests conducted on electrochemical workstation in 3.5 wt.% NaCl solution. In the three-electrode system, the sample was the working electrode, the platinum coil was the counter electrode and the saturated calomel electrode (SCE) was the reference electrode. Potentiodynamic polarization curves were started from a cathodic potential of −2000 to 3000 mV_SCE_ at 0.03 mV/s. Electrode impedance spectroscopy (EIS, CHI 760E, Shanghai Chenghua Instrument Co., Ltd., Shanghai, China) tests were proceeded under open-circuit voltage. The alternating current voltage signal varied from 0.01 Hz to 100 kHz using a 10 mV amplitude to analyze the electrode response. Zsimpwin software was used to fit the EIS experiment data.

## 3. Results and Discussion

### 3.1. Surface Properties

**Figure 1:**
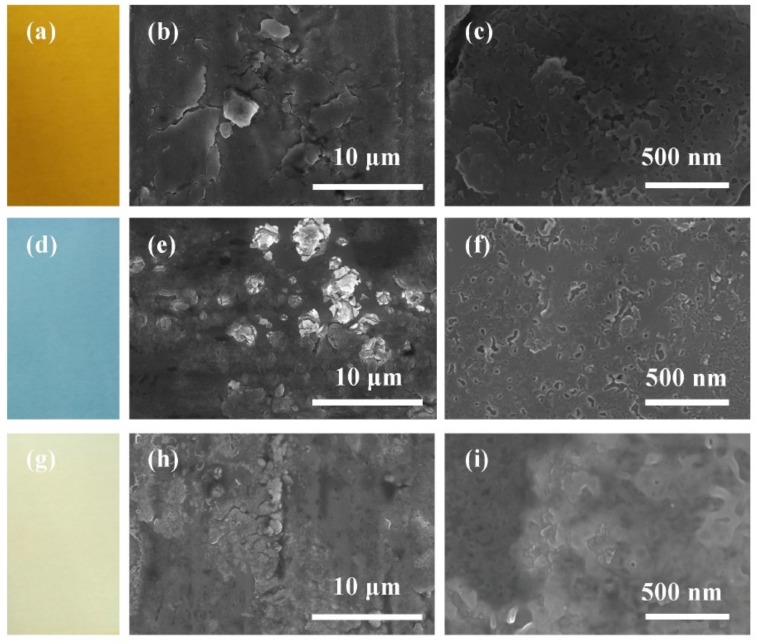
Surface colors and scanning electron microscopy (SEM) images of the surface of the oxide films formed at different voltages: (**a–c**) Ti_10_; (**d–f**) Ti_30_; (**g–i**) Ti_50_.

Figure 1 shows photos and the SEM images of CP-Ti, Ti_10_, Ti_30_, and Ti_50_ samples. As shown in Figure 1a, the color of the oxide film formed on bare material is relatively dark grey. From Figure 1b,c, it can be seen that the film of bare material has lots of holes and scratches. As shown in Figure 1d,g,j, the sample surface changed into different colors after treated by anodic oxide treatment. It is well-known that titanium and its alloy exhibit various colors because of thin film interference of oxide films [22]. As the anodic voltages increase, the colors of the oxide film change from yellowish orange to grey, which shows that the higher the anodic voltage, the thicker the oxide film produced.

From Figure 1b,c, it can be observed that many cracks and nanopores appear on the Ti_10_ surface. As shown in Figure 1e,f, the passive film formed on Ti_30_ is relatively dense, some of the nanopores are slightly smaller. Some “flower-like” structures emerge. This is in accordance with the results of the other research [23]. According to the literature [17,24], these “flower-like” structures are mainly composed of crystalline grains, and are mostly attributed to manifestation of the dielectric breakdown of the film. When the anodic potential continued to increase, as shown in Figure 1h,i, the “flower-like” structures grow intensively and spread, covering the film surface and the passive film becomes flat and dense.

**Figure 2:**
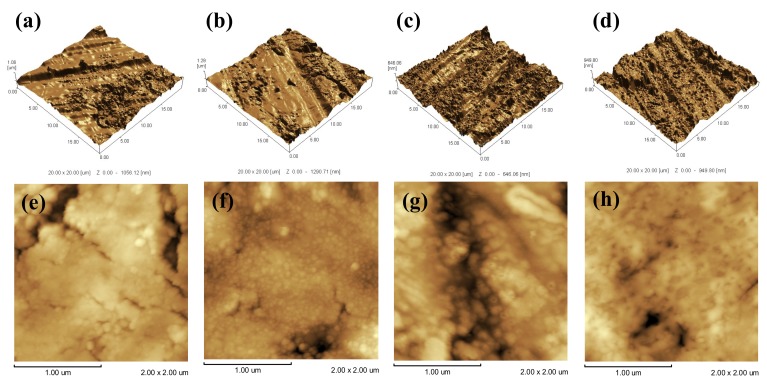
Atomic force microscope (AFM) image of three-dimensional anodic oxide film. (**a**,**e**) CP-Ti; (**b**,**f**) Ti_10_; (**c**,**g**) Ti_30_; (**d**,**h**) Ti_50_.

Figure 2 presents the AFM images of passive films produced under different anodic potential. Since the surface roughness parameter has an effect on the adhesion, adsorption and differentiation of Cl^−^ [25], it is also an important parameter affecting corrosion resistance [25]. The roughness parameter is generally labeled with R_a_, which represents the arithmetic mean of the deviation of the rough contour from the average line profile measured over the entire length [26]. The roughness measured by AFM of bare material is 121.627 nm, as shown in Figure 2a. R_a_ of the Ti_10_ is calculated to be about 106.566 nm corresponding to 3D profile as shown in Figure 2b. The Ti_30_ revealed a surface roughness R_a_ equal to 84.098 nm (Figure 2c), while Ti_50_ is quite smooth with a lower surface roughness R_a_ = 43.094 nm (Figure 2d).

Furthermore, no nanocrystalline grains can be found on the surface of CP-Ti (Figure 2e). As shown in Figure 2f, lots of nanocrystalline grains with similar shape and dimension (around 50 nm) can be observed on the surface of Ti_10_. In the case of 30 V, the randomly distributed nanoscale grains grow larger (Figure 2g). For Ti_50_, the TiO_2_ nanocrystalline grains join together and form a smooth region (Figure 2h). In summary, when the commercially pure titanium was treated at a higher potential at the potentiodynamic-aging mode, the growth of the oxide film is favored, decreasing its roughness, and increasing its flatness.

**Figure 3:**
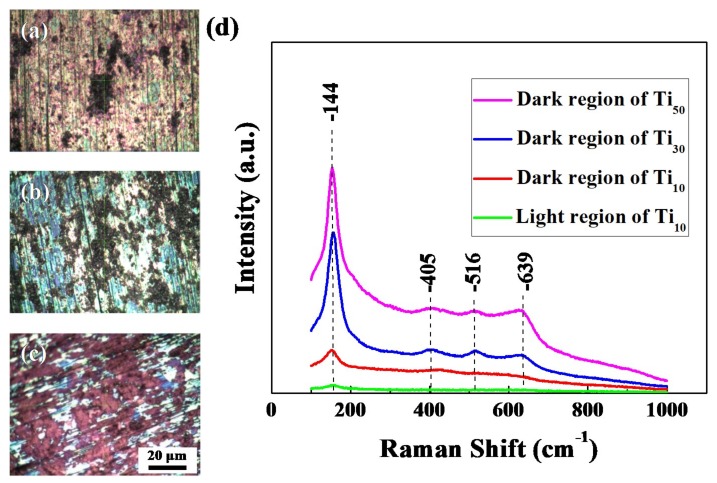
Optical microscopy pictures of passive film formed on different potentials (**a**) Ti_10_, (**b**) Ti_30_, (**c**) Ti_50_, (**d**) Raman spectrum of passive film formed on different potentials.

The crystallization of the obtained passive films was evaluated by Raman spectrum. Figure 3 shows the optical microscopy photos and Raman spectrum of passive film formed on Ti_10_, Ti_30_, and Ti_50_. The optical microscopy photos under different anodic potentials are shown in Figure 3a–c. The images of all samples have two distinguishable regions, the dark, and the light region, which means that the crystallization of the passive film is non-uniform. This phenomenon is in accordance with the results of Raman spectrum.

The Raman bands at around 144, 405, 516 and 639 cm^−1^ are in accordance with the values of the E_g_, B_1g_, A_1g_ or B_1g_ and E_g_ modes of anatase phase, respectively. Furthermore, the Raman band at about 144 cm^−1^ is the long range order of anatase phase, Raman bands at about 405, 516 and 639 cm^−1^ are the short range ones [27]. When the Raman microlaser beam is centered respectively on the dark and the light region of anodic oxide film grown on the Ti_10_, the results are shown in the spectrum red and green in Figure 3d. It is clear that a low-frequency E_g_ mode is observed in the dark region, while no Raman band can be found in the light region. The Raman band is weak and has an obvious shift towards lower wave number, which indicates that the passive film formed on Ti_10_ in dark region is composed of nanometer-scale anatase phase [28]. This phenomenon clearly implies that the dark region is more crystalline than the light region. On the Ti_30_ samples, the dark regions Raman spectra were composed of a strong band about 144 cm^−1^ and three weak bands about 405, 516, 639 cm^−1^, which are attributed to long range order and short range order of anatase phase. Additionally, when the anode voltage increased to 50 V, the Raman peaks become sharper and stronger. According to Xing et al. [23], the Raman band intensity is proportional to the film crystallinity. These results clearly show that the crystallization of passive films is enhanced by increasing the anodic potential at the potentiodynamic-aging mode.

**Figure 4:**
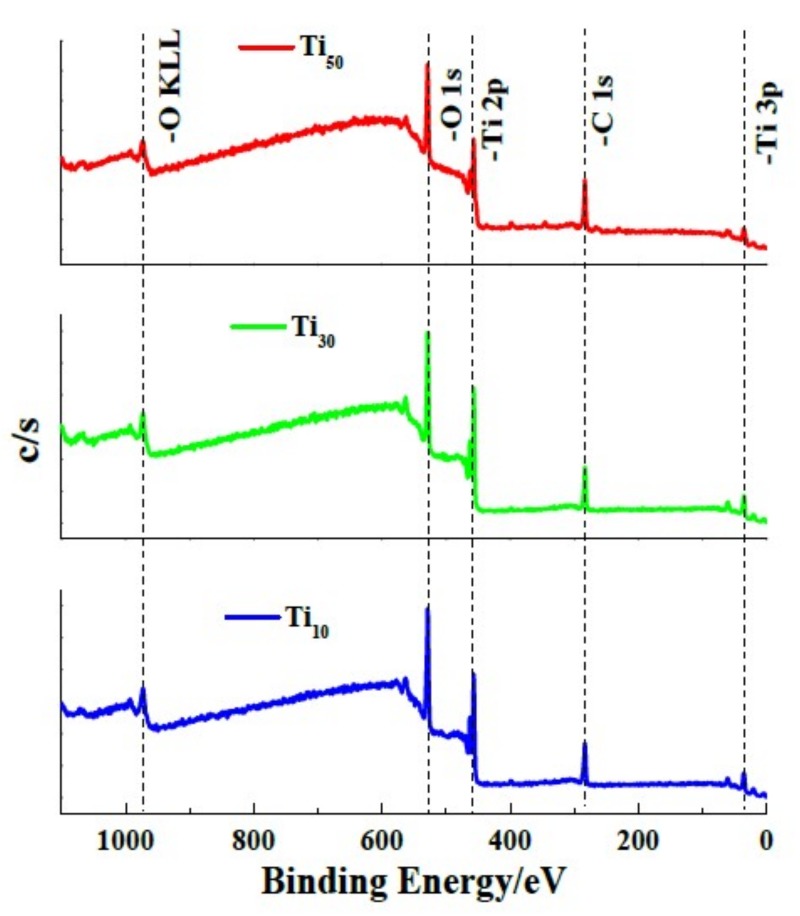
XPS survey spectra of the anodic oxide films formed on different potentials.

The surface chemical state of the anodic oxide films formed at different anodic potentials was characterized via XPS analysis. As Figure 4 shows, the resulting wide-range XPS spectrum of each specimen indicates that Ti, O and C elements in the passive film. The C 1s peak, which occurs at 284.06 eV, is attributed to the surface-contaminant hydrocarbon layer that covers the topmost surface of the samples [29].

XPS measurements were used to characterize the chemical state of passive film formed on specimens and the results are illustrated in Figure 4. By using the results from Wang et al. [30] and Jiang et al. [31], we determined the possible species in anodic oxide films, as shown in Table 1.

**Figure 5:**
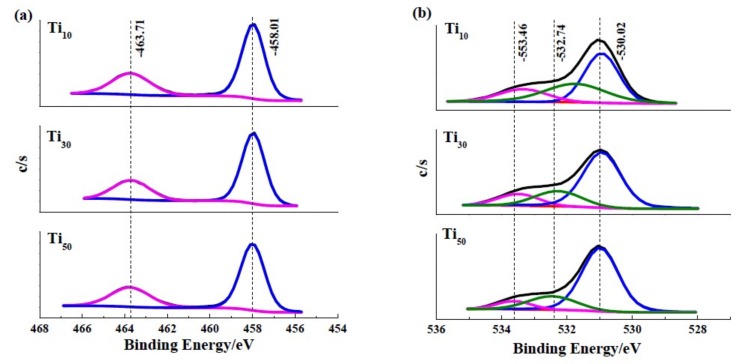
XPS analysis of the samples: (**a**) the narrow-scan spectrum of Ti 2p, (**b**) the narrow-scan spectrum of O 1s.

After deconvolution using Gaussian-Lorentzian functions, Ti 2p_3/2_ narrow-scan spectrums of the specimens, reveal a major peak at 458.01 eV corresponding to Ti^4+^ species, as shown in Figure 5a. The O 1s narrow-scan spectrums, as shown in Figure 5b, are composed of three peaks, which correspond to O^2−^, OH^−^, and adsorbed water. The O^2−^ is reported to assign to oxygen atoms in TiO_2_, and the OH^−^ and the adsorbed water may be from the hydrated titanium oxides. This phenomenon has been reported in other research [23,32]. The Ti 2p narrow-scan spectrum of all samples indicate that anodic oxidation treatment favors the transformation from lower valence states to Ti^4+^, and enhancing thermodynamically stability of the anodic oxide film. In addition these peaks reveal the outmost surface (0 nm) of all samples consist of TiO_2_, Ti(OH)_4_ and TiO_2_·nH_2_O.

The anodic potential effect on the percentage contents of O^2−^, OH^−^ and adsorbed water of the anodic oxide film are summarized in Table 2. Compared the contribution of O 1s components in Ti_10_, Ti_30_, and Ti_50_ samples, it indicates that the O^2−^ increases and the OH^−^, H_2_O decrease with increasing anodic potential. As mentioned by other research [17], the hydroxide group and adsorbed water were unfavorable for the crystallization. This result indicates that enhancing the anodic voltage has a positive effect on the formation of dehydrated and TiO_2_ dominated anodic oxide film, which is in accordance with the results of Roman spectrum.

**Figure 6:**
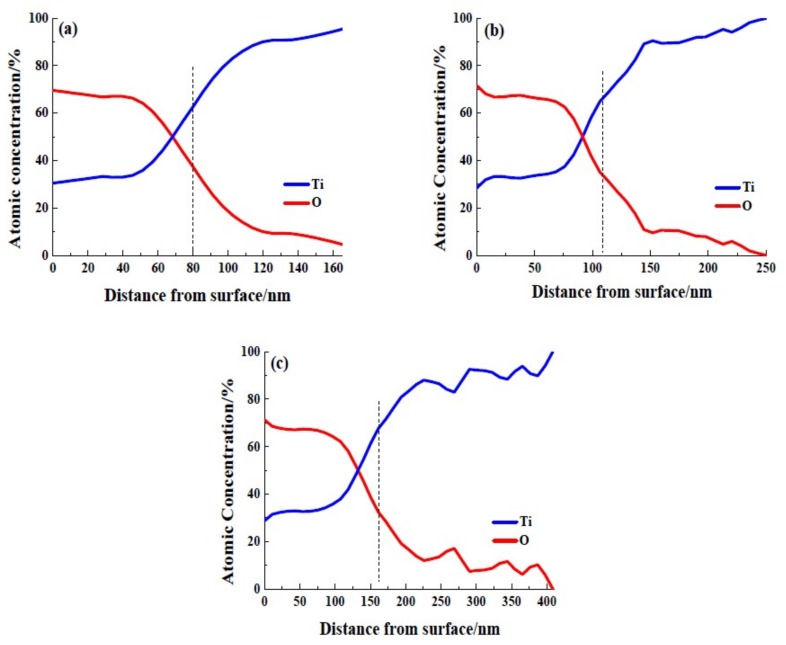
XPS depth profile of passive film and oxide film thickness vs. the anodic potential (**a**) Ti_10_; (**b**) Ti_30_; (**c**) Ti_50_.

In order to compare the thickness of the passive film formed on different anodic potentials, XPS depth profiles were carried out. Figure 6 depicts Ti and O atomic percentages varying with sputtering depth of each sample. The thickness of the anodic oxide films can be calculated by the XPS depth profiles at the point where the oxygen-atoms concentration dropped to 50% of its maximum value [33,34], in which case the value is calculated to be about 35%. Figure 6a shows that the thickness of anodic oxide film formed on 10 V is about 80 nm. Figure 6b reveals that the thickness of the anodic oxide film formed on 30 V is about 114 nm. Whereas, the thickness of the anodic oxide film formed on 50 V is about 160 nm (Figure 6c). The grown rate abstracted from Figure 6 was about 1.5 nm V^−1^, which is commonly found on other passivated metals [35].

**Figure 7:**
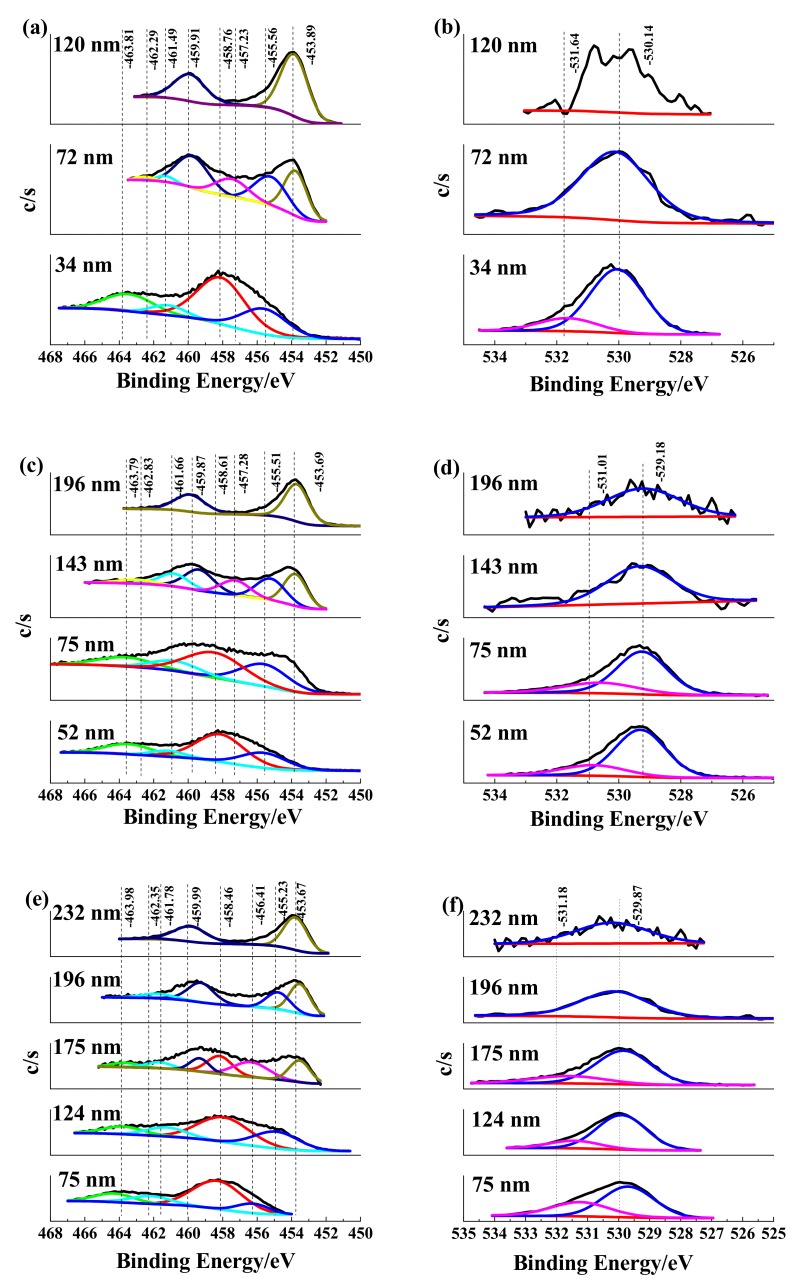
High-resolution XPS spectra of Ti 2p and O 1s obtained from Ti_10_, Ti_30_, and Ti_50_. (**a**,**b**) Ti_10_; (**c**,**d**)Ti_30_; (**e**,**f**)Ti_50_.

The XPS narrow-scan spectrum spectra recorded from surface to depth of the anodic oxide films exhibited the chemical state of Ti 2p and O 1s peaks, as shown in Figure 7a,c,f and Figure 7b,d,e respectively. For Ti_10_ sample (as shown in Figure 7a,b), the Ti2p spectra in a depth of 34 nm show the presence of Ti^2+^. Furthermore, O element existed in the form of O^2−^, OH^−^. These results reveal the anodic oxide film is consisted of TiO_2_, Ti(OH)_4_, and TiO in the depth from 0 to 34 nm. In the sputtering depth from 34 to 72 nm, Ti^3+^ and metallic Ti^0^ occurred, and the peak intensity from Ti^2+^ weakened. The XPS data suggest that the anodic oxide film in this region mainly consists of Ti_2_O_3_, TiO, and metallic Ti^0^. In a depth of 120 nm, the peaks attributed to Ti^3+^ and Ti^2+^ disappeared and the peak attributed to metallic Ti^0^ became higher. For Ti_30_ sample (as shown in Figure 7c,d), the Ti 2p spectra recorded at 52 nm is assigned to Ti^4+^ and Ti^2+^. The O 1s spectra recorded in the depth of 52 nm is composed of O^2−^ and OH^−^. The XPS data indicate that chemical compositions of the anodic oxide film are TiO_2_, TiO, and Ti(OH)_4_ in the depth of 0–52 nm. In the depth of 75 nm, the peaks attributed to Ti^4+^ weakened and the peaks due to Ti^2+^ became higher. In the depth from 75 to 143 nm, the anodic oxide film is composed of Ti_2_O_3_, TiO, and metallic Ti. In the depth of 196 nm, both Ti_2_O_3_ and TiO disappeared and only the metallic Ti^0^ is observed. For Ti_50_ sample (as shown in Figure 7e,f), the Ti 2p spectra recorded at 75 nm is composed of Ti^4+^ and Ti^3+^. The O element in this depth is O^2−^ and OH^−^. The XPS results reveal that the components in the anodic oxide film are TiO_2_, Ti_2_O_3_, and Ti(OH)_4_ in the depth from 0 to 75 nm. In the depth from 75 to 124 nm, the anodic oxide film is composed of TiO_2_, TiO, and Ti(OH)_4_. In the depth of 124–175 nm, the anodic oxide film is composed of TiO_2_, Ti_2_O_3_, Ti(OH)_4_ and metallic Ti^0^. In the depth of 175–196 nm, the major constituents of the film are TiO and metallic Ti^0^. In the depth of 232 nm, only the metallic Ti^0^ can be detected. In addition, metallic Ti^0^ can be found throughout the anodic oxide film and mainly exist near the substrate in each sample.

The XPS results also show that the anodic oxide film are mainly composed of TiO_2_, Ti_2_O_3_, TiO, metallic Ti^0^, Ti(OH)_4_, and TiO_2_.H_2_O. Therefore, the XPS analysis provides many extra pieces of evidence to support the discussions of SEM and Raman spectrum to clarify the relationship between compositions of anodic oxide films and anodic potentials.

### 3.2. Electrochemical Tests

**Figure 8:**
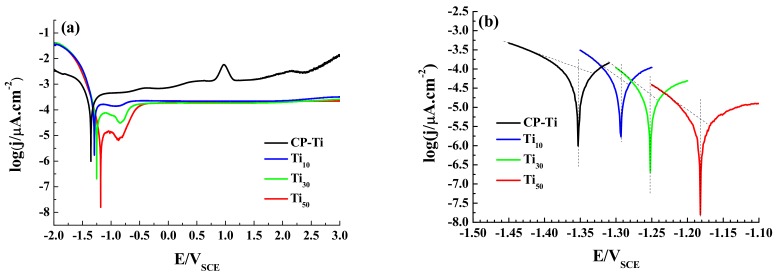
Potentiodynamic polarization curves of the CP-Ti, Ti_10_, Ti_30_ and Ti_50_ samples in 3.5 wt.% NaCl solution. (**a**) Potentiodynamic polarization curves for all samples; (**b**) Tafel extrapolation plot for all samples.

Figure 8 shows potentiodynamic polarization curves measured in 3.5 wt.% NaCl solution. In the present case, the cathodic reduction reaction is oxygen absorption reaction [36]. The chemical equation may be:

(1) $$O_{2}+2H_{2}O+4e^{-}\to 4OH^{-}$$

The corrosion potential (E_corr_) and corrosion current density (j_corr_) were obtained from the Figure 8 by Tafel extrapolation and summarized in Table 3 [37]. In order to obtain the accurate extrapolation results, anodic or cathodic branch should exhibit Tafel behavior. In addition, the extrapolation should be used at least 50 to 100 mV away from E_corr_ [38]. As Ti and its alloy do not have Tafel behavior in anodic branch [39], only the cathodic Tafel line was used to extrapolate to the E_corr_ to determine the j_corr_ in this study. E_corr_, values of –1.352, –1.297, –1.252 and –1.181 V_SCE_, and j_corr_, values of 1.258 × 10^−4^, 5.623 × 10^−5^, 1.995 × 10^−5^, and 5.623 × 10^−6^ μA·cm^−2^ were obtained for the CP-Ti, Ti_10_, Ti_30_, and Ti_50_, respectively. The higher E_corr_ and the lower j_corr_ means the lower susceptibility to corrosion [40]. The results indicate that the anodic oxidation treatment has a positive effect of improving the corrosion resistance of CP-Ti. Furthermore, as the anodic potential increases, the E_corr_ increases and j_corr_ decreases. This is a clear indication that the anodic potential has a positive effect on the corrosion resistance of CP-Ti.

**Figure 9:**
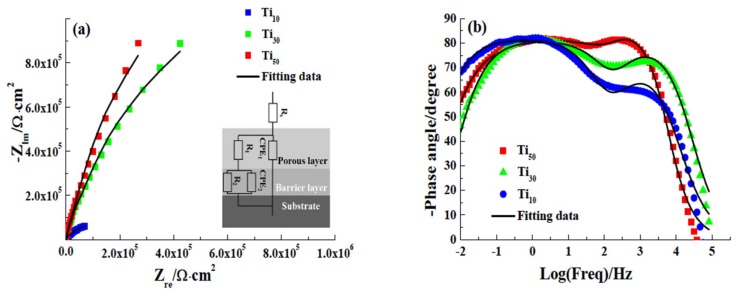
Nyquist and Bold plots of the Ti_30_ and Ti_50_ samples in 3.5 wt.% NaCl. (**a**) Nyquist plots; (**b**) Bold plots.

Electrochemical impedance spectroscopy (EIS) was used to obtain important parameters associated with the anodic oxide film, such as the charge transfer resistance, capacitance and thickness of the anodic oxide film [41]. It can be seen that Nyquist plots were characterized by flattened and incomplete semi-circles in the entire frequency range, as shown in Figure 9a. This semi-circle is associated with the charge transfer reaction occurring at the metal/electrolyte interface. The larger the diameter of the arc is, the greater the electron transfer resistance is, and the stronger the stability of the oxide film is [42]. The semi-circle increases with increasing anodic potential, which means that the anodic potential has greatly improved the corrosion resistance of CP-Ti. The Nyquist plot of the Ti_50_ is almost a straight line which indicates good insulating properties of the anodic oxide film.

Furthermore, two time constants are well distinguished in the Bode plot. According to Robin [43], the anodic oxide film formed on Ti and its alloy is a double layer structure which consists of a porous outer layer and a barrier inner layer. The EIS were fitted using an electrical equivalent circuit (EEC), in which two time constants was chosen to represent the double layer structure. In this EEC, R_S_ is the solution resistance, R_1_ and R_2_ are the charge transfer resistances of the porous layer and barrier layer. The symbol Q corresponding to a constant phase element (CPE) with varying α, stands for the possibility of a non-ideal capacitance behavior. The symbol Q corresponding to a constant phase element (CPE) with varying α, stands for the possibility of a non-ideal capacitance behavior. Q_1_ and Q_2_ are the CPE of the porous outer layer and barrier inner layer. The mathematical formulation of CPE can be obtained by Equation (2) [44]:

$$Z_{\mathrm{CPE}\left(\omega \right)}=\frac{1}{Q{\left(jw\right)}^{\alpha }}$$

where Q is the magnitude of the CPE in F·cm^−2^ s^α−1^, ω is the angular frequency (ω = 2πf) in rad·s^−1^, f is the frequency in Hz, j is the imaginary number ($j=\sqrt{-1}$), α is the CPE exponent which is adjusted between 0 and 1. For α = 1, the CPE represents an ideal capacitor; for α = 0, the CPE represents an ideal resistor; for α = 0.5, the CPE behaves a Warburg impedance with diffusion character, for 0.5 < α < 1, the CPE describes a distribution of dielectric relaxation times in the frequency domain [25,45]. The value of α is related to surface state [46].

Table 4 shows the fitted results of the impedance spectra. The Chi-squared (χ^2^) values were lower than 10^−3^ which indicates satisfactory agreement between the experimental and fitting data. R_s_ remains almost constant in all tests. R_1_ increases from 1.79 Ω·cm^−2^ for the Ti_10_ to 43.78 Ω·cm^−2^ for the Ti_30_ and further to 89.45 Ω·cm^−2^ for the Ti_50_, respectively. R_1_ was increased by two orders of magnitude through enhancing the anodic potential. Additionally, R_2_ of the Ti_10_, Ti_30_ and Ti_50_ are about 489.53, 502.56, and 512.48 Ω·cm^−2^, respectively. It is clear that the values of R_2_ are higher than the R_1_ values, indicating that the inner layer is a more compact film than the outer layer. However, R_2_ increase slowly with the increment of anodic potential, which reflects that the increase of potential has little effect on the inner layer. The value of R_1_ depends strongly on the existence of pores, channels or cracks which the solution can penetrate [47]. Combined the results of SEM, Raman spectrum, XPS, it is revealed that the surface roughness is decreased, the density and the crystallinity is increased, and the resistance of the outer porous layer significantly improved with increasing the potential.

Furthermore, the value of Q decreases with increasing applied potential. The increase of transfer charge resistances and the decreased value of Q indicated continuous growth of the anodic oxide film with the increment of potential value.

The CPE used in EEC has been converted into a pure capacitance (C) via Equation (3) [48]:

(3) $$C=\frac{{\left(Q\cdot R\right)}^{\frac{1}{\alpha }}}{R}$$

where R is the film resistance, Q is the magnitude of the CPE, α is the CPE exponent. The values of C_1_ of Ti_10_, Ti_30_, and Ti_50_ are 0.08121, 0.02611, and 0.02078 μF cm^−2^, respectively. C_2_ decreases from 0.08935 Ω·cm^−2^ for the Ti_10_ to 0.05964 Ω·cm^−2^ for the Ti_30_ and further to 0.04896 Ω·cm^−2^ for the Ti_50_, respectively. It can be seen that both C_1_ and C_2_ values decrease with the increment of potential value insulating the growth of the anodic oxide film. The thickness of the anodic oxide film can be calculated using Equation (4):

(4) $$L=\frac{\varepsilon \varepsilon_{0}}{C}$$

where ε is the relative dielectric constant of the film, ε_0_ is the dielectric constant in vacuum (8.8542 × 10^−14^ F·cm^−1^) and L denotes the film thickness. As the Raman results show that crystalline form of the film is anatase, the ε of porous outer layer can be considered as 48 [49]. Based on the XPS results, the chemical condition of barrier inner layer is amorphous titanium oxide, hence the value of ε is taken as 33 [50]. Thickness of the barrier inner layer and porous outer layer of anodic oxide films formed at different potentials are shown in Table 5, it is clear that the porous outer layer thickness is larger than the barrier inner layer. Moreover, the potential has a greater influence on the thickness of the outer porous layer.

The EIS results demonstrate that increasing anodic potential positive affects the protective properties of the anodic oxide films formed on CP-Ti. Increasing the potential can increase the thickness of the anodic oxide film and the charge transfer resistance, and the effect on the outer layer is more obvious.

### 3.3. Discussion

**Figure 10:**
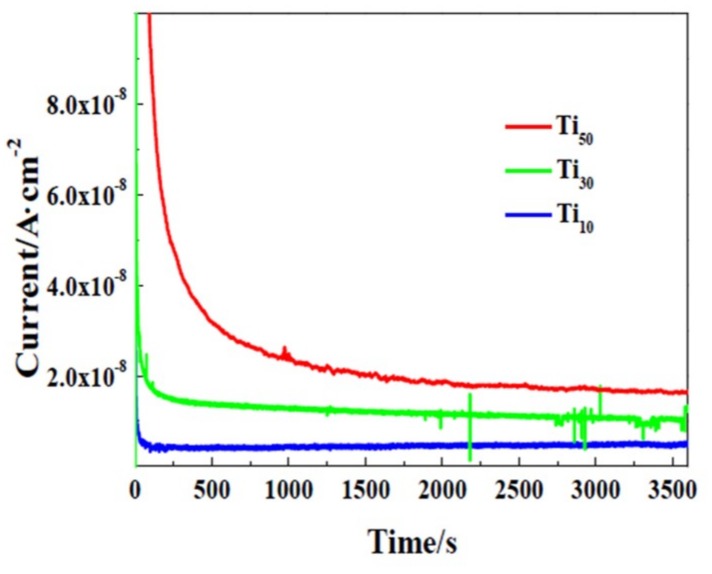
Chrono-amperometric curves of CP-Ti anodization under potentiodynamic-aging mode at 10, 30 and 50 V.

I–t curves of CP-Ti under potentiodynamic-aging mode at 10, 30 and 50 V are shown in Figure 10. It can be seen from the curves that the current density is very large at the first stage. Subsequently, the current density decreases dramatically during the first few seconds. Finally it remains at steady state until the end of the anodic treatment. It can be indicated that the oxide film has been formed in only a few seconds and the current fluctuations may be related to the oxygen evolution reaction (OER) [51]. Moreover, it can be also found that after the initial drop, the steady state current density is much larger for CP-Ti anodization at higher anodic potential. Additionally, the larger current density at higher anodic potential means that the film growth is higher [52].

**Figure 11:**
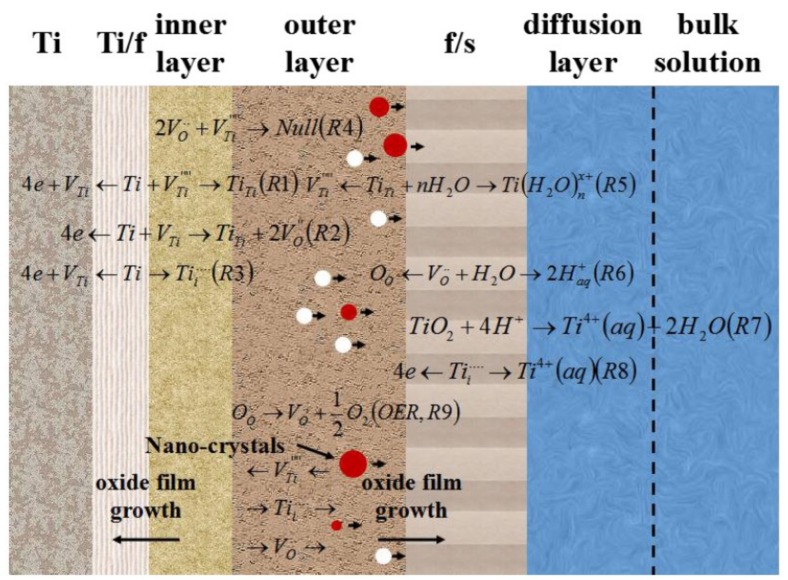
Schematic illustration of the growth process of oxide films.

**Figure 12:**
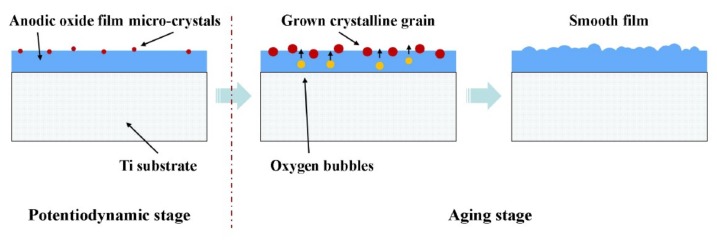
Schematic illustration of the crystallization process of oxide films under different potential.

Based on the results mentioned above, the growth and crystallization process of passive films under potentiodynamic-aging mode are shown in Figure 11 and Figure 12. According to our previous studies [53], the oxide film of titanium is an n-type semiconductors, which indicates the defects in the oxide film are donors, such as oxygen vacancies and titanium interstitials [54]. The formation energy of oxygen vacancies and titanium interstitials is 2.7 and 4.7 eV, respectively [55]. Therefore, oxygen vacancies are considered to be dominant donors. However, titanium interstitials is also under consideration in the present study.

These point defects transport through the oxide film and undergo defect reactions at the Ti/oxide film (Ti/f) and film/solution (f/s) interfaces. According to Veluchamy et al. [56], the transport and reaction of these point defects determine the growth of the oxide film. The point defect model (PDM) was used to describe the growth process of oxide films on Ti surfaces and can be schematically illustrated in Figure 10 and R1–R8 (Figure 11).

The equations are expressed in Kröger-Vink notation [57], where the V_Ti_ represents Ti vacancy, V_Ti_^’’’’^ repesents Ti cation vacancy, V_O_^••^ represents O vacancy, Ti_Ti_ represents Ti in Ti site on the cation sublattice, Ti_i_^••••^ represents Ti cation interstitial, O_O_ represents oxide ion in oxide site on the anion sublattice, Ti^4+^(aq) represents Ti^4+^ in solution, Ti(H_2_O)_n_^4+^ repesents Ti and hydrated titanium complexes, respectively. The electrical field of the oxide film enables the defects through the film at room temperature [57,58].

R1–R3 happen at the Ti/inner layer interface. R1 expresses the submergence of cation vacancies, V_Ti_^’’’’^ into the Ti lattice. It results in the formation of Ti_Ti_ in the oxide lattice and a vacancy V_Ti_ into the Ti lattice. R2 represents Ti reacting with V_Ti_ which generates Ti_Ti_ and V_O_^••^. It results in the movement of the Ti/f toward the Ti side. R3 corresponds to metal Ti generating V_Ti_^’’’’^ and V_Ti_. R5–R8 take place at the outer layer film/solution interface (f|s). R5 represents the formation of V_Ti_^’’’’^ and Ti(H_2_O)_n_^4+^, which are soluble. V_Ti_^’’’’^ is transferred by the electric field toward the Ti|f where they are annihilated towards the Ti side (R1). R6 indicates the absorption of O^2−^ into V_O_^••^ resulting in O_O_. R7 represents the dissolution of the oxide film. In addition, the dissolution of the oxide film is necessary for the oxide film to attain steady-state. Cation-vacancy annihilation (via R4) leads to V_O_^••^/V_Ti_^’’’’^ interactions, which are driven by the electrostatic attraction between high concentrations of oppositely charged defects [59]. R9 corresponds to the oxygen evolution reaction.

The addition of R1 and R5 or R2 and R6 result in the Ti_i_^••••^ and V_Ti_^’’’’^ eliminated, yields:

(5) $$\mathrm{Ti}\to {\mathrm{Ti}}^{4+}\left(aq\right)+v_{\mathrm{Ti}}+4e^{-}$$

which indicates metal Ti dissolution. The barrier inner layer is a semi-permeable membrane [54]. The addition of R3 and R8, result in the oxygen vacancy eliminated, yields:

(6) $$\mathrm{Ti}+\chi H_{2}O\to Ti_{Ti}+\chi O_{O}+e^{-}$$

which corresponds to the growth of the barrier layer. V_O_^••^ generated at f/s via R2 and annihilated at Ti/f via R6. By means of hopping mechanism, the O_O_ ions are transferred through V_O_^••^ toward the Ti/f. Meanwhile, V_O_^••^ is transferred towards the f/s via the electric field and the concentration gradient [54], which determines barrier layer growth towards into the metal. The species in the solution does not participate in the growth of the barrier layer. This result is in accordance with the XPS results that the barrier layer contains only Ti and O elements. The outer layer forms by the hydrolysis of H_2_O. As a result, the outer layer comprises oxide, hydroxide and hydrated oxide. The growth rate of the anodic oxide film is related to the concentration of oxygen vacancies which depends on applied potential [3]. Therefore, the higher the anodic potential, the thicker film oxide film thickness.

The crystallization stage of anodic titanium oxide films under different potentials are shown in Figure 11. As mentioned by other research [60,61], the crystallization of the of anodic oxide film is considered to be a thermally-induced, slow evolving process, and promoted by enhancing the oxide potential.

For Ti_10_ sample, at the end of grown stage, the current density is relatively small, the film crystallization occurs at a very slow speed, numerous micro-crystals randomly emerge at the beginning of titanium anodization and grow to small crystalline grains in the following process. For the Ti_30_ sample, the current density is relatively large, the oxygen bubbles are formed and released from the surface due to the OER, and the films breakdown is enhanced. As reported by Dyer et al. [62], crystallization is promoted by breakdown of amorphous anodic oxide films because the breakdown can enhance the local current density. As a result, numerous of TiO_2_ micro-crystals grow quickly, and form bigger crystalline grains. When the titanium treated at 50 V, at the end of the grown stage, the current density is very large and the OER is very high frequency. Therefore, the micro-crystals are quickly formed. With the anodizing time prolonged, these micro-crystals join together and form a smooth region.

## 4. Conclusions

The effect of anodic potential on passive films in 0.5 M H_2_SO_4_ solution has been investigated using SEM, AFM, XPS, potentiodynamic polarization curves and EIS.

The SEM and AFM results show that the anodic potential has a positive effect on the characteristics of the passive film. With increasing anodic potential, the surface micromorphology became more compact and lower roughness. XPS analysis revealed that the thickness of the anodic oxide film increases with increasing anodic potential. The potentiodynamic polarization curves revealed that the E_corr_ shifted towards more positive values and corrosion current density j_corr_ values decreased with increasing anodic potential.

## Figures and Tables

**Table 1:** Energies of XPS-peaks of standards.

Ti 2p_3/2_ (eV)				O 1s (eV)		
Ti	Ti^2+^	Ti^3+^	Ti^4+^	O^2−^	OH^−^	H_2_O
453.6	455.9	457.3	458.7	530.2	532.5	533.4

**Table 2:** The contribution of O 1s components on the surface of Ti_10_, Ti_30_ and Ti_50_.

Samples	The Contribution of O 1s Components (%)		
	O^2−^	OH^−^	H_2_O
Ti_10_	73.65	21.88	4.47
Ti_30_	78.03	17.59	4.38
Ti_50_	84.09	12.99	2.92

**Table 3:** Electrochemical parameters of the CP-Ti, Ti_10_, Ti_30_ and Ti_50_ in 3.5 wt.% NaCl solution at room temperature.

Sample	E_corr_/V_SCE_	j_corr_ (μA cm^−2)^
CP-Ti	−1.352	1.258 × 10^−4^
Ti_10_	−1.297	5.623 × 10^−5^
Ti_30_	−1.252	1.995 × 10^−5^
Ti_50_	−1.181	5.623 × 10^−6^

**Table 4:** Fitting data of electrode impedance spectroscopy (EIS) for the Ti_30_ and Ti_50_.

Samples	R_s_ (kΩ·cm^2^)	Q_1_/10^−5^ (Ω^−1^·cm^−2^s^α^)	α_1_	Q_2_/10^−5^ (Ω^−1^·cm^−2^s^α^)	α_2_	R_1_ (kΩ·cm^2^)	R_2_ (kΩ·cm^2^)	χ^2^ (10^−4^)
Ti_10_	1.02	8.5501	0.92	2.5680	0.67	1.79	489.53	3.47
Ti_30_	1.12	2.5727	0.89	1.7535	0.64	43.78	502.56	2.87
Ti_50_	1.08	1.9826	0.93	1.3068	0.59	89.45	512.48	2.67

**Table 5:** Capacitance and thickness of porous outer layer and barrier inner layer for titanium anodized films formed under different potentials.

Samples	C_1_ (μF·cm^−2^)	C_2_ (μF·cm^−2^)	L_1_ (nm)	L_2_ (nm)
Ti_10_	0.08121	0.08935	52.33	32.68
Ti_30_	0.02611	0.05964	162.75	49.86
Ti_50_	0.02078	0.04896	204.48	59.64
